# Phytoremediation Performance with Ornamental Plants in Monocultures and Polycultures Conditions Using Constructed Wetlands Technology

**DOI:** 10.3390/plants13071051

**Published:** 2024-04-08

**Authors:** José Luis Marín-Muñiz, Irma Zitácuaro-Contreras, Gonzalo Ortega-Pineda, Aarón López-Roldán, Monserrat Vidal-Álvarez, Karina E. Martínez-Aguilar, Luis M. Álvarez-Hernández, Sergio Zamora-Castro

**Affiliations:** 1 Academy of Sustainability and Regional Development, El Colegio de Veracruz, Xalapa 91000, Veracruz, Mexico; jmarin@colver.info (J.L.M.-M.); izitacuaro@yahoo.com (I.Z.-C.); gopc30@gmail.com (G.O.-P.); aaron.rolescolar@gmail.com (A.L.-R.); monserrat.vidal@gmail.com (M.V.-Á.); kemartineza.ddrs22@colver.info (K.E.M.-A.); lmalvarezh.ddrs22@colver.info (L.M.Á.-H.); 2 Faculty of Engineering, Construction and Habitat, Universidad Veracruzana, Bv. Adolfo Ruiz Cortines 455, Costa Verde, Boca del Río 94294, Veracruz, Mexico

**Keywords:** clean technology, plant cultures, environmental impact index, sustainability, wastewater treatment

## Abstract

The assessment of constructed wetlands (CWs) has gained interest in the last 20 years for wastewater treatment in Latin American regions. However, the effects of culture systems with different ornamental species in CWs for phytoremediation are little known. In this study, some chemical parameters such as total suspended solids (TSS), chemical oxygen demand (COD), phosphate (PO_4_-P), and ammonium (NH_4_-N) were analyzed in order to prove the removal of pollutants by phytoremediation in CWs. The environmental impact index based on eutrophication reduction (EI-E) was also calculated to estimate the cause-effect relationship using CWs in different culture conditions. *C. hybrids* and *Dieffenbachia seguine* were used in monoculture and polyculture (both species mixed) mesocosm CWs. One hundred eighty days of the study showed that CWs with plants in monoculture/polyculture conditions removed significant amounts of organic matter (TSS and COD) (*p* > 0.05; 40–55% TSS and 80–90% COD). Nitrogen and phosphorous compounds were significantly lower in the monoculture of *D. seguine* (*p* < 0.05) than in monocultures of *C. hybrids,* and polyculture systems. EI-E indicator was inversely proportional to the phosphorous removed, showing a smaller environmental impact with the polyculture systems (0.006 kg PO₄^3−^ eq removed) than monocultures, identifying the influence of polyculture systems on the potential environmental impacts compared with the phytoremediation function in monocultures (0.011–0.014 kg PO₄^3−^ eq removed). Future research is required to determine other types of categories of environmental impact index and compare them with other wastewater treatment systems and plants. Phytoremediation with the ornamental plants studied in CWs is a good option for wastewater treatment using a plant-based cleanup technology.

## 1. Introduction

Constructed wetlands (CWs) are a sustainable technology for wastewater treatment. This strategy is based on natural wetland processes for the removal of contaminants, including physical, chemical, and biological routes, but in a more controlled environment compared with natural ecosystems [1]. This ecotechnology involves three important components: porous filter media, microorganisms, and vegetation. CWs are used to remove pollutants from rainwater, municipal, industrial, and agricultural wastewater with the phytoremediation processes [2,3,4].

The different mechanisms involved in wastewater treatment are mainly biodegradation by microbial processes (via aerobic/anaerobic mechanisms), plant uptake (phytoremediation), sorption, photodegradation, and volatilization. The ability of CWs to remove organic matter, nitrogen and phosphorus compounds, emerging pollutants (antibiotics, human and veterinary pharmaceuticals, personal-care products, etc.), pesticides of agricultural runoff, and coliforms from wastewater has been studied extensively around the world, especially in the U.S.A. and Europe, resulting in high-efficiency levels [5,6,7] or in simultaneous nutrient removal and power generation [8,9]. In addition, CWs are cost-effective, and maintenance costs are less when compared to conventional treatment systems [10].

According to the flow of water, CWs can be free water surfaces (FWS) or subsurface CWs (SS-CWs). FWS CWs have areas of open water and are similar in appearance to natural marshes. In SS-CWs, the water circulates from a substrate or filter media, and the flow direction can be vertical (VF-CWs) or horizontal (HF-CWs). HF-CWs typically employ gravel beds where the vegetation is planted. The water is kept below the surface of the bed horizontally from the inlet to the outlet [1,11]. VF-CWs can receive and treat heavy loads, leading to high load reductions due to the feeding regime that is applied. The wetland surface is flooded with wastewater, which infiltrates the system via gravity, favoring high levels of oxygen transfer within the filter media, leading to aeration and microbial activity but also minimizing clogging phenomena [11,12].

Achieving sustainability of the decentralized use of wastewater is a challenge that developing countries are currently facing. The Latin American and Caribbean region is a heterogeneous territory with a wide variety of environmental conditions, and CWs seem to be an adequate solution for the region’s wastewater challenges. Rodríguez-Domínguez et al. [13] analyzed the use of CWs in such zones, where the results showed a generally good performance for organic matter and nitrogen removal but a low performance for phosphorous removal. In Mexico, García-García et al. [14] reported that the interest in CWs is growing exponentially, particularly in academic institutions. Consequently, published works are mostly on experimental wetlands, although there are a few experienced groups devoted to producing technology and providing the training needed to apply CWs.

Considering the water scarcity faced in arid and semiarid regions, alternative water supplies like treated water can thus be used for irrigation of green spaces, toilet water, floor washing, orchards, etc. Several barriers need to be overcome to increase the adoption and utilization of CW technology in Mexico and other regions, including the lack of knowledge regarding this technology, scarce technical information, environmental education, the government’s concentration on constructing wastewater treatment plants solely in urban areas, and the optimal design according to the substrate of filter media and the plants [15,16].

Regarding the filter media used in CWs, a recent review described that gravel and sand are the most used substrates worldwide [17]; however, other alternatives, such as zeolite or pyrite, have been investigated. In some studies, the use of zeolite and pyrite was compared with common mineral materials (gravel). Subsequently, it was found that zeolite and pyrite filters were more efficient for nitrogen removal compared to gravel [18,19] due to the adsorption capacity in the porous media. Marín et al. [7] evaluated porous river rock and tepezyl materials, showing important effects on the removal of pollutants. The cost associated with these mineral materials can avoid the use of CWs as a low-cost alternative in sites with limited economic resources, which is common in tropical regions.

Therefore, it is important to consider the use of reused material, such as plastic waste, with rough or porous characteristics that may favor the formation of microbial families for the removal of pollutants.

In tropical regions, a characteristic of some CWs is the use of local ornamental plants (*Heliconia* sp. *Zantedeschia aethiopica*, *Canna* genus) for removing pollutants [3,4,10,15,16]. Most of the studies in these CWs have been done using ornamental plant monocultures. However, polyculture of ornamental plants may form floristical arrangements with better aesthetic value than monoculture systems [4].

CWs with mixed plant cultures may have a more effective distribution of their root biomass and may provide a habitat for more diverse microbial organisms, enhancing the release of root exudates that favor the uptake of inorganic compounds compared to CWs with only monoculture plants [3,4,7].

Liang et al. [20]. reported that there is a significantly lower ammonium removal in the first year of operation of a mixed wetland (*C. indica*, *C. flabelliformis*, *P. australis*, *Pennistum purpureum*, and *H. littoralis*) when compared to a monoculture, but later on the removal is significantly higher for the mixed culture. Carrillo et al. [21] detected that CWs planted with polycultures reduce eutrophication conditions (5–17%), but inversely, polycultures had the highest environmental impact in global warming than monocultures (1.5–9 times) compared to those planted in monocultures.

Thus, there is a need for a better understanding of the use of ornamental vegetation in CWs to achieve optimal design and operation in ecotechnology and to clarify optimal functional design with different ornamental culture plants in the treatment process.

Likewise, in addition to the functionality of CWs in terms of contaminant removal, it is also important to analyze the environmental impact of the technology, which recent studies demonstrate using life cycle analysis and its different categories that imply indices of climate change, depletion of abiotic resources or eutrophication indices (EI-E), among others [22]. The EI-E is one of the most widely used. Therefore, its analysis is pertinent in terms of evaluating the potential impact on the environment and health of the CWs under study and comparing monoculture and polyculture systems.

Considering the previously mentioned, the objective of this study is to evaluate the wastewater treatment with domiciliary CWs planted with ornamental vegetation in monoculture and polyculture conditions and use plastic residues as a substitute for filter media. To the best of our knowledge, there are no environmental studies of CWs planted with polycultures of this combination of species and their environmental impact compared with monoculture systems.

## 2. Results and Discussion

### 2.1. Physic-Chemical Parameters

Data regarding wastewater before entering the CWs and after the treatment period are described in Table 1. The pH in water was an average value of 7.4 ± 0.3 at the influent, while the output was the same value detected in the monoculture and polyculture treatments (7.6). Ekhlasur et al. [23] reported that the presence of plants in CWs regulates the pH (~7.5), improving plant growth conditions. The DO was 1.1 ± 0.3 mg L^−1^ at the input, while at the output, the concentrations were between 3.8 and 3.9 for systems with monoculture of plants and 4.1 ± 0.3 mg L^−1^ in systems in polyculture treatment. This increase could be due to the radial oxygen release carried out by the plants [24]. On the other hand, the water temperature was 20.2 ± 0.5 at the influent, while the output in the monoculture and polyculture treatments was almost 2 °C lower (18.2–18.6 °C), probably due to the shadow generated by the plants, favoring a decrease in the temperature in the water.

### 2.2. Plant Growth

**Figure 1:**
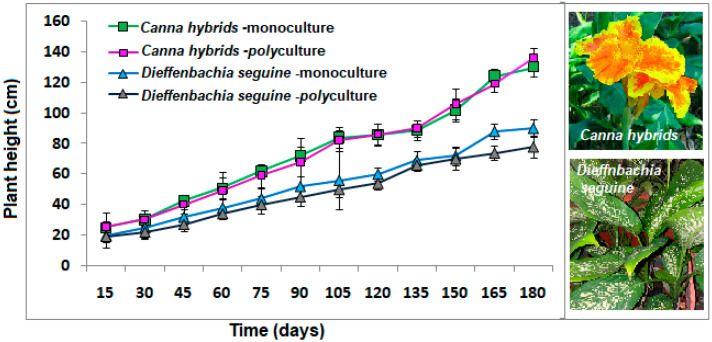
Plant growth of ornamental plants in this study.

Growth of the *Canna h.* (Figure 1) in the 180 days of the study tended to be similar in monoculture or polyculture conditions (*p* = 0.311) (without possible interspecific competition among species in the same space in polyculture conditions) but higher (125–130 cm) than *Dieffenbachia s*. (65–80 cm), which is derivative of the nature of the species since the former can reach up to 2.2 m in height and grows well in waterlogged conditions with frequent exposure to sun sites [25]. In the case of *Dieffenbachia s*., it is not a common wetland species; such plants grow best under partial shade of trees (1–2 m) [26], but its adaptation was shown, although slower (Figure 1).

In addition, the cultivation of the species *Canna h.* (also called achira, sagú, or flag, depending on the country) and the extraction of its starch are important activities for the economy of different countries such as Colombia, Taiwan, and China [27]. The easy adaptation to wastewater treatment systems of this species leads to the consideration of it as an ornamental attractive option in this type of technology due to its flowering, the additional use of the flower for seedling production, and the use of its components for medicinal aspects [26,28].

For *Dieffenbachia s*., its production would be important for those who manage the system since it is a common and popular exotic indoor ornamental plant [27]. In monoculture conditions, this species grows better (2–6 cm) than in polyculture characteristics (Figure 1) but without significant differences (*p* = 214). Considering these advantages, plus the ability to function as a removal of pollutants (phytoremediation), they are now an important alternative in CW systems. Future research about ornamental plants in CWs should compare the growth of the species in both conditions: greywater and clean water.

### 2.3. Pollutant Concentrations and Removals

Several studies have shown the positive effects of plants as pollutant removal in constructed wetlands compared with systems without vegetation, where the pollutant removal in experiments without plants oscillated within 15–30% lower than in experiments done with vegetation [4,28,29,30]. With this information, we did not consider the implementation of experiments without vegetation necessary; we only compared the removal of pollutants in single-family domiciliary CWs with monoculture and polyculture of ornamental plants.

The concentrations of contaminants at the entrance of the system changed for all the measured parameters; this was a result of the variations in the discharge of greywater since the dishwashing that originates organic residues or the discharge of laundry water, contains significant amounts of phosphorus, is not a daily discharge, while personal bathing or household cleaning wastewater is more constant.

**Figure 2:**
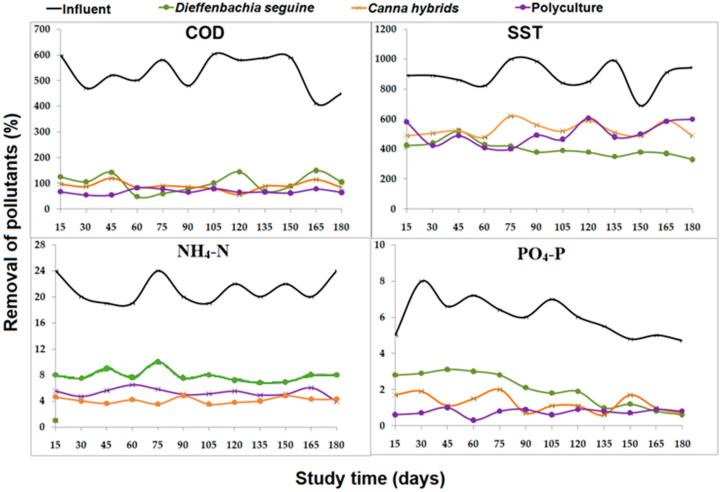
Temporal changes of COD SST, NH_4_-N, and PO_4_-P concentrations during the study.

During the study, the COD concentrations upon entering the system remained in a range of 400–500 mg/L, while at the exit of the system, these decreased in a range between 50 and 150 mg/L in the three types of treatments (Figure 2). The average concentration of COD was 530.4 ± 90.1 mg/L, while the outputs ranged from 68–102 mg/L. Low concentrations observed indicated the removal of such parameters in a range of 80% to 87% in both monoculture and polyculture systems, without statistical differences between the two cultures of plants on removals (*p* = 0.811; Table 2). The detected is related to the observed in Figure 1, where the growth of both species in the two plant culture was statistically similar, but with adaptation to CWs condition, situation that favored the COD reduction, alluded to the oxygen supply provided by plants via their parenchymal system, which favors the development of vital microbial community responsible for the process of removal of the organic matter [29,30,31], related with the DO detected in Table 1, similar for the three treatments (3.8–4.1 mg/L).

The removals obtained revealed that the water was considered acceptable in quality, according to references of the Mexican National Water Commission [32], which has been monitoring the water quality of rivers since 1974 based on COD, BOD_5,_ and total solids parameters, and according to the maximum permissible limits for discharges into rivers and streams (150 mg/L, monthly average) established limits by Mexican regulations [32].

Regarding the concentration of total suspended solids during the influent, these were in a range of 700–1000 mg/L, while during the 180 days, it was similar within monoculture and polyculture systems (400–600 mg/L; Figure 2). The TSS average in the influent was 889.4 ± 99.3 mg/L (Table 2). In effluents, the values were 401.2, 530.3, and 502.7 mg/L in monocultures of *Dieffenbachia seguine*, *Canna hybrids*, and polyculture CWs, respectively. These concentrations represent 54.9, 40.4, and 43.5 of removals, without differences between the two cultures of plants (*p* = 0.625; Table 2), showing the importance of the systems for the TSS reduction. The observation confirms the importance of combining ornamental species to promote the aesthetics of the system without compromising the growth of plants (Figure 1) or the phytoremediation.

A study using cattail plants revealed that these species are able to decrease TSS levels; however, it is recommended to apply a longer residence time to improve the rate of contaminant removal [33]. Russell [34] indicates that water with acceptable quality must be between 76–150 mg/L, but in this study, the inlet concentration was 889.4 ± 99.3 mg/L, which was reduced when passing through the systems (401–530), showing that the treated water still had high concentrations of TSS, despite removals of 40–55%, an important level using only CWs. Other studies [3,4,26] also reported low TSS removals related to the short maturation time of the system and the low hydraulic retention time, as could have occurred in this study.

For NH_4_-N and PO_4_-P, the range of concentrations in the influent were 20–25 mg/L and 5–8 mg/L, respectively. At the effluent, they were 4–10 mg/L and 0–3 mg/L, respectively (Figure 2), except for ammonia (average 80.8 ± 8.7 mg/L) and phosphate (average 86.7 ± 10.6 mg/L), the pollutant measured did not show statistically (*p* > 0.05) different removal rate between polycultures and monocultures with *Canna hybrids* (75–80%; *p* > 0.05), compared with the removal in monoculture CWs of *Dieffenbachia seguine* (63–68%; *p* < 0.05). Luo et al. [35] indicated that plant communities with various growth forms reduced the intensity of interspecific competition, increased functional diversity, and greatly enhanced the ability to consume nitrogen and phosphorous compounds.

These results provide evidence about the importance of combining ornamental plants in CW designs. N-NH_4_ parameter in Mexico is not regulated; it was compared with data from the Taiwan Environmental Protection Agency, where they describe that 0.5 mg/L of the compound is permissible for the protection of aquatic life [36], so more polishing is still required of wastewater using only the CWs, the addition of hybrid CWs should be an option to increase the ammonium removal.

For PO_4_-P data, it can be noted that the detected ion concentration exceeded the limits set by the USEPA [37], which establishes a maximum of 0.05 mg/L ion in water bodies to prevent eutrophication problems. Similar to ammonium problems. The addition of hybrid CWs should be an option to increase the PO_4_-P removals.

These results showed the importance of the use of CWs with ornamental plants in domiciliary conditions to avoid discharges in rivers when other conventional systems are not installed. This ecotechnology provides a good aesthetic appearance and removes both organic and inorganic compounds from wastewater. Other authors [38,39,40,41] indicated that CWs with polyculture of ornamental plants also provide biodiversity enhancement and better social adoption. Vera-Puerto et al. [42] reported that ornamental plants should be used carefully (reviewing climate conditions) to ensure the best quality and esthetics for their implementation in CWs. 

### 2.4. Analysis of the Environmental Impact in CWs with Different Culture Plants

**Figure 3:**
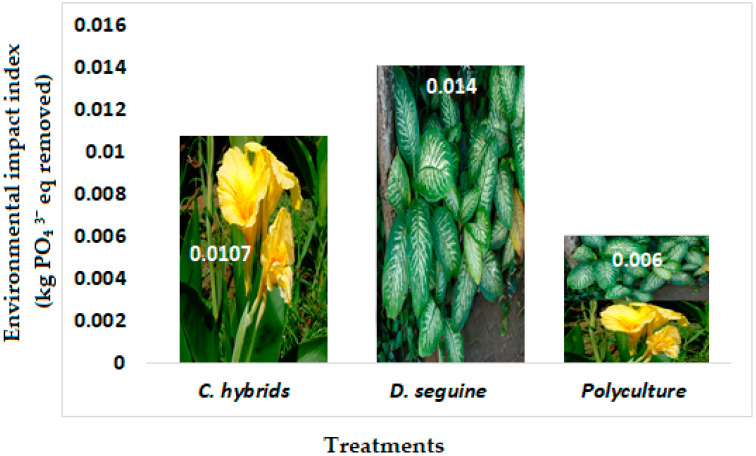
Comparative of the potential environmental index for each culture plant treatment.

The EI-E index is inversely proportional to phosphorous removal efficiency: a higher index represents a lower removal efficiency [21] (Figure 3). In this study, *C. hybrids* (0.011 kg PO₄^3−^ eq) and *D. seguine* (0.014 kg PO₄^3−^ eq) monocultures presented the highest EI-E index, related to low phosphorous removal efficiencies of 60–80% (Figure 3). Polyculture systems showed ~87% of removal, presenting a eutrophication index of 0.006 kg PO₄^3−^ eq removed, better than monoculture treatments, related to the described growth (Figure 1) and removal efficiency in polycultures mesocosms (Figure 2, Table 1). These results were smaller than the data detected using activated sludge wastewater treatment (0.072–0.340) [43]. The PE index observed in polyculture systems in this study was similar to the one detected in CWs combined with other types of ornamental species (*Cyperus papyrus* + *Zantedeschia aethiopica*) in Chile [21].

According to the results, it is important to highlight that the plant or culture of species is a vital factor to consider for the best design and environmental performance of CWs [4,44]. The choice of vegetation-composing CWs should not be based only on the plant removal capacity (Table 1) since the introduction of invasive plants can be an ecological problem [45]. Vegetation plays a vital role in bringing necessary physical effects in order to remove and retain pollutants and nutrient cycling; with the detected, it is necessary to strengthen networking among scientists, stakeholders, industrialists, governments, and non-government organizations in the use of phytoremediation with CWs for wastewater problems [46,47,48]. On the other hand, the support or filter medium is also worth mentioning, as it could influence the wastewater treatment. In this study, the support medium was tezontle mixed with plastic residues, which presents a good phosphorus removal efficiency and anchoring of the vegetation. Plastic residues and polyculture of ornamental vegetation are approaches that have the potential to enhance the beautiful appearance of CWs and reduce building costs (decrease the economic and environmental impact), as the most used substrates in CWs (gravel, sand, zeolite) are much more expensive (10–35 USD/m^3^) [17,49].

## 3. Materials and Methods

### 3.1. Description Techniques and Study Area

**Figure 4:**
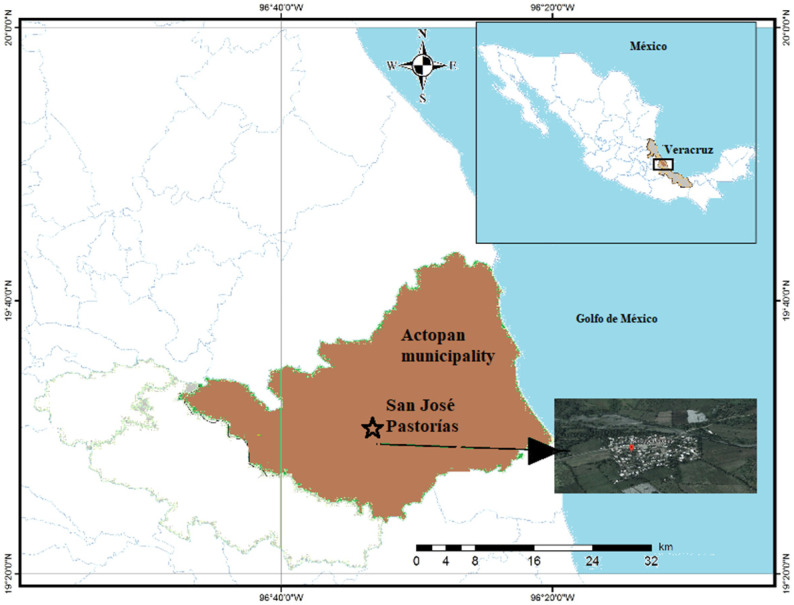
Location of the study site (San José Pastorías, Actopan; red point).

**Figure 5:**
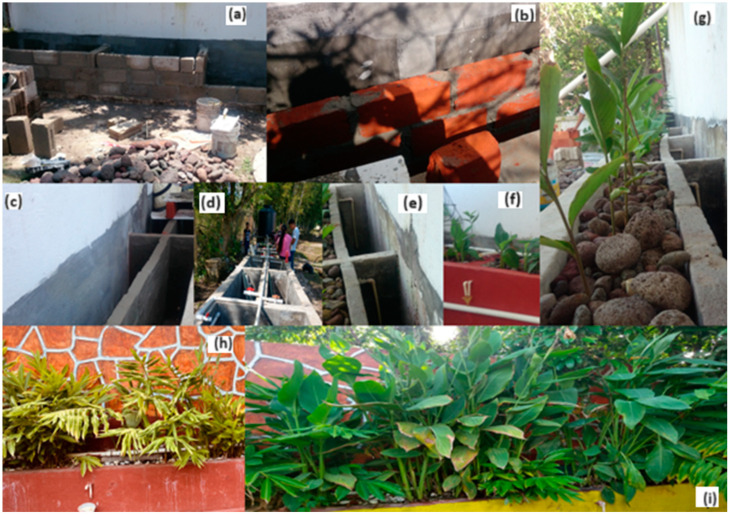
Construction of domiciliary treatment wetland for wastewater at the household level. (**a**) CWs cell construction, (**b**) red blocks used in CWs, (**c**) CWs cell structures, (**d**,**e**) installation of the pipeline, (**f**,**g**) planted species, (**h**) systems after three months of construction, (**i**) systems after six months of construction.

The household level was evaluated in San José Pastorías (Municipality of Actopan), Veracruz, Mexico (−96°57′08″ N and 19°55′83″ S) (Figure 4). The average annual rainfall fluctuates between 1200 and 1650 mm. The annual average temperature varies between 18 °C and 36 °C, with an average of 27 °C. Six wetland mesocosms were constructed in the backyard of a single-family house (1.5 m long, 0.23 m width, 0.60 m depth). The construction was made with building bricks (Figure 5a,b). The waterproofing was made with concrete (cement) (Figure 5c–e).

The size of the CWs was determined based on the area. For example, Rivas et al. [50] have reported that the overall system surface area is 3.4 m^2^/people equivalent, taking as an example the wetland built-in Pastorías, Actopan, analyzed in this study. This was made for three people. Wastewater was collected in a septic tank (1000 L). Later, a plastic mesh was put at the end of the hose in order to trap large suspended solids. From the tank, a tube distributed the water to each cell, and each mesocosm had a tap fitted with a hydraulic retention time (HRT) of 3 days (flow rate: 17.4 mL/min).

The CWs were filled with different layers of filter materials (porous river rock and plastic residues); in this case, the porous river rock (average porosity 50%) was collected from the local river (Topiltepec) in the Pastorías community. A rock layer of 15 cm with a particle size of approximately 12 cm was placed at the bottom of each cell in order to avoid clogging. Then, a medium layer (35 cm) with plastic residues (bottlenecks, bottle caps, and the rough base of bottles). Finally, the upper layer was porous river rock with a particle size of approximately 4 cm, placed with water flow at a distance of 10 cm below the surface (Figure 5g). After the cells were filled with the filter media, two of them were planted with *Dieffenbachia seguine* (4 plants of 20 cm), two more with *Canna hybrids* (monoculture cells with four plants of 20 cm [15–18 cm] and the remaining two cells were the polyculture systems, planted with two plants of *D. seguine* and two plants of *C. hybrid* (Figure 5h–i). All CWs were kept flooded for six weeks, and the wastewater was diluted with tap water for vegetation adaptation. Then, they were continuously fed with only wastewater from a single-family residence.

### 3.2. Sampling and Analytical Methods

The water samples (350 mL) were taken from the influent and effluent of each mesocosm every 15 days (*n* = 12 with a replicate for each treatment) during the 180 days of study. The water was analyzed for chemical oxygen demand (COD), nitrogen as ammonium (N-NH_4_), and phosphate (PO_4_-P) (Table 2), measured according to standard methods [51,52]. Dissolved oxygen (DO), total suspended solids (TSS), pH, and water temperatures were measured with a multiparameter instrument (HANNA^®^ instruments Mexico, CDMX, Mexico, model: HANNA03077). The average concentrations of the analyzed physicochemical parameters in the wastewater influents (Ci) and effluents (Ce) were used to calculate the removal efficiencies of each CWs parameter according to Equation [1,7]: Removal efficiency = [(Ci − Ce)/Ci] × 100%. Individual plant height was measured every 15 days using a measuring tape.

### 3.3. Phytoremediation Performance of Environmental Impact of CWs with Different Culture Plants

To compare the effects of the wastewater treatment in different plant culture conditions and the environmental impact, the Environmental impact index was used [43,53], based on the total removal of phosphorous (kg eq/kg P removed) and using the eutrophication potential factor (EPF): 0.42 and 0.095 for NH_4_ and PO₄^3−^, respectively.

EI-E = ⅀ EPF × *m*

where EI-E = eutrophication potential in the water (expressed in kg equivalents of PO₄^3−^) and *m* is the mass in kilograms of the substance emitted to water.

This eutrophication can be defined as the enrichment of nutrients such as nitrogen and phosphorus in the aquatic environment. This phenomenon produces an increase in biomass production, deoxygenation of water, and thus fish mortality, among others [43,53].

### 3.4. Statistical Analysis

Statistical analyses were performed with SPSS version 22 for Windows. One-way analysis of variance (ANOVA) was used to investigate the phytoremediation effect of plants in monoculture and polyculture CWs. A 5% significance level was used to determine differences among treatments and among the growth of plant culture.

## 4. Conclusions

Phytoremediation using CWs is cost-effective and does not rely on operational energy inputs, demonstrating their feasibility for single-family homes, especially for developing countries. The use of local material as filter media and the use of local ornamental plants are important components to consider prior to the implementation of this technology. Ecology systems with ornamental flowering plants are an excellent option due to the phytoremediation effect of plants and their aesthetic appearance in the garden. *Canna hybrids* and *Dieffenbachia seguine* used in this study showed that organic matter was removed from both cultures of plants. However, monocultures of C. hybrids and polyculture systems were more efficient for nitrogen and phosphorous removals. The EI-E indicator was inversely proportional to the phosphorous removed, showing the smaller environmental impact of polyculture systems and identifying the influence of polyculture systems on the potential environmental impacts compared with the vegetation in monocultures. CWs with polycultures of ornamental plants at the household level are a good option for greywater treatment. Thus, it is essential to implement environmental legislation and/or public policy regarding the issue of wastewater treatment in order to mitigate pollution problems using CWs with ornamental plants in rural and urban areas.

## Figures and Tables

**Table 1:** Physico-chemical parameter concentrations are at the input and outputs of the treatment wetlands.

Parameters		Monoculture CWs		Polyculture CWs
	Influent	*Dieffenbachia seguine*	*Canna hybrid*	*Dieffenbachia seguine + Canna hybrid*
pH (pH units)	7.4 ± 0.3	7.6 ± 0.3	7.6 ± 0.3	7.6 ± 0.4
DO (mg L^−1^)	1.1 ± 0.3	3.8 ± 0.4	3.9 ± 0.6	4.1 ± 0.3
Temperature (°C)	20.2 ± 0.5	18.6 ± 0.6	18.2 ± 1.4	18.6 ± 0.2

Values are average ± Standard error.

**Table 2:** Removal efficiencies (%) of pollutants in constructed wetlands.

Parameters		Monoculture CWs				Polyculture CWs	
		*Dieffenbachia seguine*		*Canna hybrid*		*Dieffenbachia seguine* + *Canna hybrid*	
	Inflow Concentration (mg/L)	Outflow Concentration (mg/L)	Removal (%)	Outflow Concentration (mg/L)	Removal (%)	Outflow Concentration (mg/L)	Removal (%)
TSS	889.4 ± 99.3	401.2 ± 52.1	54.9 ± 9.1 ^a^	530.3 ± 20.3	44 ± 9.2 ^a^	502.7 ± 22.6	43.5 ± 4.9 ^a^
COD	530.4 ± 90.1	102.1 ± 18.2	80.8 ± 7.3 ^a^	90.1 ± 9.6	83.0 ± 5.7 ^a^	68.6 ± 16.3	87.1 ± 4.4 ^a^
NH_4_-N	21.3 ± 5.4	7.8 ± 1.6	63.4 ± 8.3 ^b^	5.2 ± 0.8	75.6 ± 6.4 ^a^	4.1 ± 0.7	80.8 ± 8.7 ^a^
PO_4_-P	6.02 ± 0.9	1.9 ± 0.2	68.4 ± 9.6 ^b^	1.2 ± 03	80.1± 9.3 ^a^	0.8 ± 0.1	86.7 ± 10.6 ^a^

Values are average ± standard error, different letters indicate significant differences between the rows at 5% significance level. TSS: total suspended solids, COD: chemical oxygen demand, PO_4_-P: phosphate, and NH_4_-N: ammonium.

## Data Availability

The data presented in this study are available on request from the corresponding author.
